# Genetic background may contribute to the latitude-dependent prevalence of dermatomyositis and anti-TIF1-γ autoantibodies in adult patients with myositis

**DOI:** 10.1186/s13075-018-1617-9

**Published:** 2018-06-08

**Authors:** Joanna E. Parkes, Simon Rothwell, Alexander Oldroyd, Hector Chinoy, Janine A. Lamb, The Myositis Genetics Consortium (MYOGEN), Ingrid E. Lundberg, Frederick W. Miller, Robert G. Cooper, William E. Ollier, Peter K. Gregersen, Jiri Vencovsky, Katalin Danko, Vidya Limaye, Albert Selva-O’Callaghan, Pedro M. Machado, Michael G. Hanna, Hazel Platt, Øyvind Molberg, Olivier Benveniste, Timothy Radstake, Jan De Bleecker, Boel De Paepe, Britta Maurer, Leonid Padyukov, Terrance P. O’Hanlon, Lucy R. Wedderburn, Christopher Denton, Herman Mann, David Hilton-Jones, Patrick Kiely, Paul H. Plotz, Mark Gourley, Galina Marder, Neil J. McHugh, Zoe E. Betteridge

**Affiliations:** 1 Centre for Epidemiology, Division of Population Health, Health Services Research & Primary Care, Faculty of Biology, Medicine and Health, Manchester Academic Health Science Centre, University of Manchester, 2.706 Stopford Building, Oxford Road, Manchester, M13 9PT UK; 2 Centre for Musculoskeletal Research, Division of Musculoskeletal & Dermatological Sciences, Faculty of Biology, Medicine and Health, Manchester Academic Health Science Centre, University of Manchester, Manchester, UK; 3 Arthritis Research UK Centre for Epidemiology, Centre for Musculoskeletal Research, University of Manchester, Manchester Academic Health Science Centre, Manchester, UK; 4 National Institute for Health Research Manchester Biomedical Research Centre, Central Manchester University Hospitals NHS Foundation Trust, Manchester Academic Health Science Centre, University of Manchester, Manchester, UK; 5 Department of Rheumatology, Salford Royal NHS Foundation Trust, Manchester Academic Health Science Centre, Salford, UK

**Keywords:** Dermatomyositis, Polymyositis, Anti-TIF1-γ, Anti-Mi-2, Latitude, Ultraviolet light

## Abstract

**Background:**

The prevalence of dermatomyositis (DM) versus DM and polymyositis (PM) combined has been shown to be negatively associated with latitude. This observation has been attributed to increasing exposure to ultraviolet (UV) light towards the equator. In this study, we investigated whether differing genetic background in populations could contribute to this distribution of DM.

**Methods:**

Case data derived from the MYOGEN (Myositis Genetics Consortium) Immunochip study (*n* = 1769) were used to model the association of DM prevalence and DM-specific autoantibodies with latitude. Control data (*n* = 9911) were used to model the relationship of human leucocyte antigen (HLA) associated with DM autoantibodies and DM or PM single-nucleotide polymorphisms (suggestive significance in the Immunochip project, *P* < 2.25 × 10^− 5^) in healthy control subjects with latitude. All variables were analysed against latitude using ordered logistic regression, adjusted for sex.

**Results:**

The prevalence of DM, as a proportion of DM and PM combined, and the presence of anti-transcription intermediary factor 1 (anti-TIF1-γ) autoantibodies were both significantly negatively associated with latitude (OR 0.96, 95% CI 0.95–0.98, *P* < 0.001; and OR 0.95, 95% CI 0.92–0.99, *P* = 0.004, respectively). HLA alleles significantly associated with anti-Mi-2 and anti-TIF1-γ autoantibodies also were strongly negatively associated with latitude (OR 0.97, 95% CI 0.96–0.98, *P* < 0.001 and OR 0.98, 95% CI 0.97–0.99, *P* < 0.001, respectively). The frequency of five PM- or DM-associated SNPs showed a significant association with latitude (*P* < 0.05), and the direction of four of these associations was consistent with the latitude associations of the clinical phenotypes.

**Conclusions:**

These results lend some support to the hypothesis that genetic background, in addition to UV exposure, may contribute to the distribution of DM.

**Electronic supplementary material:**

The online version of this article (10.1186/s13075-018-1617-9) contains supplementary material, which is available to authorized users.

## Background

The idiopathic inflammatory myopathies (IIMs) are a spectrum of rare and heterogeneous autoimmune diseases causing inflammation and weakness of skeletal muscle. Major clinical subgroups are polymyositis (PM) and dermatomyositis (DM). The relative prevalence of PM versus DM varies between populations, and it appears to be dependent on latitude; as latitude increases, the relative prevalence of DM (i.e., DM/DM and PM combined) decreases [1, 2]. This effect has been attributed to higher ultraviolet (UV) radiation levels increasing the likelihood of the development of DM over PM towards the equator, and also increasing the prevalence of the DM-specific autoantibody anti-Mi-2 [2–4]. UV radiation is thought to play a role in DM owing to skin changes being more prominent on the sun-exposed parts of the face, neck and shoulders, reports of photoaggravation of skin lesions and increased cutaneous photosensitivity compared with healthy control individuals [5, 6]. UV radiation is used in phototherapy of skin conditions such as psoriasis as well as being a major risk factor for skin cancer owing to its suppression of the immune system. UV radiation affects the immune system in various ways, including DNA damage via production of ROS, induction of cytokine signalling and induction of T-regulatory cells [7]. Although thought to be linked to the development of some autoimmune disorders, it is unclear how UV radiation contributes to the pathogenesis of conditions such as DM [2].

Notably, latitude and UV radiation intensity do not perfectly correlate, and UV exposure may depend on the behaviour of individuals, such as whether they work indoors or outdoors. It is possible that the observed differences in DM, PM and anti-Mi-2 prevalence with latitude could additionally be influenced by other factors, such as the different genetic backgrounds of populations. The most strongly associated single-nucleotide polymorphism (SNP) outside the human leucocyte antigen (HLA) gene complex for the PM subgroup in a recent IIM Immunochip study (rs2476601 in *PTPN22*) [8] demonstrates a correlation with latitude, with increasing risk allele frequency from southern to northern Europe [9]. Similarly, some HLA allele frequencies are known to vary with latitude, such as HLA-B27, associated with ankylosing spondylitis, which is absent in populations around the equator but increases to 40% in populations in the far north and the Arctic [10].

The aim of this study was to explore the associations of PM and DM prevalence and autoantibody frequencies with latitude. In particular, we investigated whether these “latitude-dependent” associations could instead be attributed to genetic background rather than environmental factors by analysing the distribution of PM and DM risk SNPs in healthy populations.

## Methods

### Association of relative prevalence of PM and DM, and DM autoantibody frequency with latitude in a large myositis cohort

Data from the IIM Immunochip study [8] were used to model the association of relative prevalence of PM or DM and DM-specific autoantibody frequency with latitude. Of the DM-specific autoantibodies, only anti-transcription intermediary factor 1-γ (anti-TIF1-γ) (97 positive, 1345 tested) and anti-Mi-2 (83 positive, 1471 tested) were analysed, because only a limited number of patients were anti-SAE- (*n* = 29) or anti-MDA5-positive (*n* = 18). Autoantibodies were tested in various centres using immunoprecipitation, line blot or enzyme-linked immunosorbent assays. Geographical latitude was assigned according to the approximate latitude of the recruitment centre which contributed samples to the MYOGEN (Myositis Genetics Consortium) Immunochip study as a proxy for latitude of origin and disease onset [8]. Data were available from 1769 cases (914 PM and 855 DM) from 12 countries worldwide (Additional file 1). All individuals included in the study were Caucasian. Juvenile cases were excluded because some contributing centres collected only juvenile DM cases, and this may have introduced bias into the latitudinal associations.

### Analysis of association of PM and DM single-nucleotide polymorphism risk alleles with latitude in healthy control subjects

The risk allele frequencies of ten SNPs associated with PM or DM (suggestive significance threshold of *P* < 2.25 × 10^− 5^) in the Immunochip study [8] were analysed for their association with latitude using the control individuals from the study (*n* = 9911). Recruitment centre information was not available for some control individuals; for these samples average latitude of the contributing country was used (*see* Additional file 1 for details). U.S. control samples lacking recruitment centre information were excluded owing to the large range of possible latitudes.

### Analysis of association of HLA alleles with DM-specific autoantibodies with latitude in healthy control subjects

HLA alleles associated with DM-specific autoantibodies also were analysed for their association with latitude in the Immunochip control individuals (*n* = 9911). In the Immunochip data HLA-DRB1* 07:01 is strongly associated with anti-Mi-2, and HLA-DQB1*02 is strongly associated with anti-TIF1-γ (*P* = 4.36 × 10^− 12^ and *P* = 2.11 × 10^− 11^ respectively [11], Rothwell S: Personal communication, in preparation). All associations were modelled using ordered logistic regression, adjusted for sex. Analysis was carried out using STATA version 13.1 (StataCorp, College Station, TX, USA) [12].

## Results

### Relative prevalence of DM and frequency of anti-TIF1-γ autoantibodies are significantly negatively associated with latitude in adult myositis

In ordered logistic regression analysis using the IIM Immunochip case data (*n* = 1769), the relative prevalence of DM (versus PM and DM combined) had a significant association with latitude (OR 0.96, 95% CI 0.95–0.98, *P* < 0.001) (Additional file 2). The presence of anti-TIF1-γ autoantibodies in the cases tested (*n* = 1345) was negatively associated with latitude (OR 0.95, 95% CI 0.92–0.99, *P* = 0.004). However, the presence of anti-Mi-2 autoantibodies in the cases tested (*n* = 1471) was not significantly associated with latitude (Table 1).

**Table 1:** Association of latitude with dermatomyositis prevalence, dermatomyositis-specific autoantibody and associated human leucocyte antigen allele frequency

Variable	Associations with latitude, adjusted for sex	
	OR (95% CI)	*P* value
Relative prevalence of DM	0.96 (0.95, 0.98)	< 0.001
Anti-Mi-2	0.99 (0.97, 1.03)	0.927
Anti-TIF1-γ	0.95 (0.92, 0.99)	0.004
HLA-DRB1*07:01 (associated with anti-Mi-2)	0.97 (0.96, 0.98)	< 0.001
HLA-DQB1*02 (associated with anti-TIF1-γ)	0.98 (0.97, 0.99)	< 0.001

*DM* Dermatomyositis, *HLA* Human leucocyte antigen, *TIF1-γ* Transcription intermediary factor 1

All variables were analysed against latitude in an ordered logistic regression analysis, adjusted for sex. Relative prevalence of DM was calculated as the frequency of DM/the frequency of polymyositis (PM) and DM combined. The proportion of autoantibodies was calculated from all adult PM and DM cases tested for that particular autoantibody (*n* = 1471 tested for anti-Mi-2, *n* = 1345 tested for anti-TIF1-γ), derived from case data in the Immunochip study (*n* = 1769). HLA allele frequencies were calculated from the Immunochip control data (*n* = 9911) for DM-specific autoantibody associations in the Immunochip data (HLA-DRB1*07:01 with anti-Mi-2 and HLA-DQB1*02 with anti-TIF1-γ, *P* = 4.36 × 10^− 12^ and *P* = 2.11 × 10^− 11^, respectively [11]; Rothwell S: Personal communication, in preparation)

### PM and DM SNP risk alleles are significantly associated with latitude in healthy populations

In ordered logistic regression analysis using data from the Immunochip study controls (*n* = 9911), the frequency of four of eight PM-associated SNP risk alleles showed a significant association with latitude (*P* < 0.05); the three most significant associations were positively associated. One of the two DM-associated SNPs was significantly negatively associated with latitude (Table 2).

**Table 2:** Association of polymyositis- or dermatomyositis-associated single-nucleotide polymorphisms with latitude

Variable	Immunochip associations and *P* values for subgroups			Associations with latitude, adjusted for sex	
	Reported gene	Polymyositis	Dermatomyositis	OR (95% CI)	*P* value
PM associations					
rs2476601	*PTPN22*	7.90 × 10^−11^	0.05	1.08 (1.05, 1.11)	< 0.001
rs4690220	*SLC26A1* | *IDUA*	7.47 × 10^−6^	0.03	1.02 (1.01, 1.02)	0.001
rs7956536	*UBE3B* | *MMAB*	3.66 × 10^−6^	0.67	1.01 (1.00, 1.02)	0.004
rs17799348	*FAM167A* | *BLK*	4.13 × 10^−6^	0.32	0.99 (0.98, 0.99)	0.035
rs1420095	*IL18R1*	6.16 × 10^−6^	0.87	0.99 (0.98, 1.01)	0.334
rs2286896	*NAB1*	3.76 × 10^−6^	0.58	0.99 (0.98, 1.01)	0.434
rs9905921	*LOC728073* | *RPL38*	2.01 × 10^−6^	0.10	0.99 (0.99, 1.01)	0.748
rs7535818	*RGS1*	1.37 × 10^−5^	0.14	0.99 (0.99, 1.01)	0.897
DM associations					
rs4702698	*ROPN1L* | *ANKRD33B*	0.5	4.49 × 10^−6^	0.99 (0.98, 0.99)	0.012
rs570676	*PRR5L* | *TRAF6*	0.01	6.23 × 10^−7^	0.99 (0.98, 1.00)	0.081

*DM* Dermatomyositis, *PM* Polymyositis

All variables were analysed against latitude in an ordered logistic regression analysis, adjusted for sex. Single-nucleotide polymorphisms (SNPs) identified as having suggestively significant *P* values (*P* < 2.25 × 10^−5^) for either PM or DM in the Immunochip study [8] were used. SNP risk allele frequencies were calculated from the Immunochip study control data (*n* = 9911)

### HLA alleles associated with DM-specific autoantibodies are significantly associated with latitude in healthy control individuals

In ordered logistic regression analysis using control data from the IIM Immunochip study (*n* = 9911), HLA alleles associated with the DM-specific autoantibodies anti-Mi-2 and anti-TIF1-γ were strongly negatively associated with latitude (OR 0.97, 95% CI 0.96–0.98, *P* < 0.001; and OR 0.98, 95% CI 0.97–0.99, *P* < 0.001, respectively).

## Discussion

In this study we analysed the associations between latitude and DM relative prevalence and DM-specific autoantibody frequencies in the largest IIM cohort to date. In addition, we analysed the association of latitude with PM- or DM-associated SNP risk allele frequencies and HLA alleles associated with DM autoantibodies in healthy populations. The results confirmed that the proportion of DM increases towards the equator, with a strong negative association with latitude. Furthermore, we showed, for the first time, to our knowledge, that DM-specific anti-TIF1-γ autoantibody frequency was significantly negatively associated with latitude along with HLA alleles associated with anti-TIF1-γ and anti-Mi-2. However, we failed to replicate the increase in the presence of anti-Mi-2 autoantibodies towards the equator which was reported in a previous study (*P* = 0.0349) [2]. The association of anti-Mi-2 with surface UV radiation intensity in that study was more significant (*P* = 0.0196) than the association with latitude and later replicated in another study of UV radiation [2, 3]. In addition, exposing keratinocytes to UV radiation has been shown to increase the expression of Mi-2 protein [13], supporting the hypothesis that UV radiation may be associated with production of anti-Mi-2 autoantibodies.

In the analysis of PM- and DM-associated non-HLA SNP risk allele frequency, we found that half of the SNPs analysed were significantly associated with latitude and four of five were consistent with the latitude associations of PM or DM prevalence (positive for PM and negative for DM). Effect sizes were modest, but the ORs had tight CIs and were statistically significant.

This study has some limitations, including the assumption that the patients’ location at disease onset and place of birth would be similar to the location of the recruitment centres. The study was limited to one ethnic group because the genetic association study was performed only in Caucasian samples. In addition, samples were collected within a limited range of latitudes, and the cases and controls were not from exactly the same populations. Studies with collection of disease onset and place of birth data along with UV exposure data in a larger multi-ethnic IIM and control cohort spread over a greater range of latitudes could elucidate whether latitude associations are due to population genetic background or environmental factors.

## Conclusions

Overall, these results lend some support to the hypothesis that different genetic backgrounds of populations may contribute to the increased prevalence of DM toward the equator, in addition to, or instead of, increasing UV radiation exposure levels.

## Additional files

Additional file 1: **Table S1.** Immunochip study data included in analyses. Assigned latitude and numbers of PM, DM and controls at each centre included in the study. (DOCX 25 kb)

Additional file 2: **Figure S1.** The association of relative dermatomyositis (DM) prevalence, frequency of DM-associated autoantibodies, HLA alleles and single-nucleotide polymorphisms with latitude. Two-way linear prediction plot with 95% CIs was produced using STATA version 13.1 software. Data for DM as a proportion of PM and DM (*n* = 1769), frequency of anti-TIF1-γ in all samples tested (*n* = 1345) and frequency of anti-Mi-2 in all samples tested (*n* = 1471) are derived from the Immunochip study [8]. Frequencies of HLA-DQB1*02, HLA-DRB1*07:01, rs4702698 and rs570676 are derived from the Immunochip control data (*n* = 9911). (TIF 2203 kb)

## Abbreviations

DM: Dermatomyositis
HLA: Human leucocyte antigen
IIM: Idiopathic inflammatory myopathy
MYOGEN: Myositis Genetics Consortium
PM: Polymyositis
SNP: Single-nucleotide polymorphism
TIF1-γ: Transcription intermediary factor 1-γ
UV: Ultraviolet
