# Supplementary material for: Genetic background may contribute to the latitude-dependent prevalence of dermatomyositis and anti-TIF1-γ autoantibodies in adult patients with myositis
Source: Arthritis Res Ther. 2018 Jun 8;20:117. doi: 10.1186/s13075-018-1617-9 (PMC5994128; doi:10.1186/s13075-018-1617-9)
Supplement: Supplementary file 1 — Table S1. Immunochip study data included in analyses. Assigned latitude and numbers of PM, DM and controls at each centre included in the study. (DOCX 25 kb) [file 13075_2018_1617_MOESM1_ESM.docx]

**Additional Table 1. Immunochip data included in analyses**

| **Country** | **Centre** | **Latitude assigned** | **Number of individuals** | | | | | | |
| --- | --- | --- | --- | --- | --- | --- | --- | --- | --- |
|  |  |  | **Adult PM** | **Adult DM** | **Controls** | **Anti-Mi2 Positive** | **Anti-Mi2 Negative** | **Anti-TIF1γ Positive** | **Anti-TIF1γ Negative** |
| Australia | Royal Adelaide Hospital | (-)34.9212 | 57 | 19 | 0 | 4 | 60 | 0 | 64 |
| Belgium | Ghent University Hospital | 51.0247 | 4 | 2 | 0 | 0 | 6 | 0 | 6 |
| Czech Republic | Charles University, Prague | 50.0872 | 102 | 122 | 0 | 16 | 176 | 21 | 170 |
| France | Pitié-Salpêtrière Hospital, Paris | 48.8372 | 6 | 11 | 0 | 0 | 16 | 0 | 16 |
| Hungary | University of Debrecen | 47.5537 | 128 | 60 | 0 | 2 | 92 | 0 | 94 |
| Italy(RAF) | Not available | 45.4069 | 0 | 0 | 785 | n/a | n/a | n/a | n/a |
| Netherlands | University Medical Center Utrecht | 52.0864 | 16 | 13 | 2,003 | 0 | 14 | 0 | 0 |
| Norway | Oslo University Hospital | 59.9402 | 25 | 32 | 0 | 3 | 47 | 0 | 50 |
| Poland | Not available | 52.6404 | 0 | 0 | 523 | n/a | n/a | n/a | n/a |
| Switzerland | University Hospital Zurich | 47.3769 | 2 | 1 | 0 | 0 | 0 | 0 | 0 |
| Sweden | Karolinska University Hospital | 59.3521 | 143 | 90 | 1,928 | 8 | 177 | 11 | 174 |
| Spain | Vall d'Hebron Hospital, Barcelona | 41.4280 | 25 | 39 | 389 | 1 | 60 | 4 | 57 |
| UK | Various | 52.5026 | 370 | 290 | 4,283 | 39 | 597 | 41 | 593 |
| US | NIH, Bethesda, MD | 39.0032 | 36 | 164 | 0 | 10 | 143 | 20 | 24 |
| US | North Shore University Hospital, New York | 40.7778 | 0 | 12 | 0 | 0 | 0 | 0 | 0 |
