# Supplementary figures and images for: Genetic background may contribute to the latitude-dependent prevalence of dermatomyositis and anti-TIF1-γ autoantibodies in adult patients with myositis
Source: Arthritis Res Ther. 2018 Jun 8;20:117. doi: 10.1186/s13075-018-1617-9 (PMC5994128; doi:10.1186/s13075-018-1617-9)

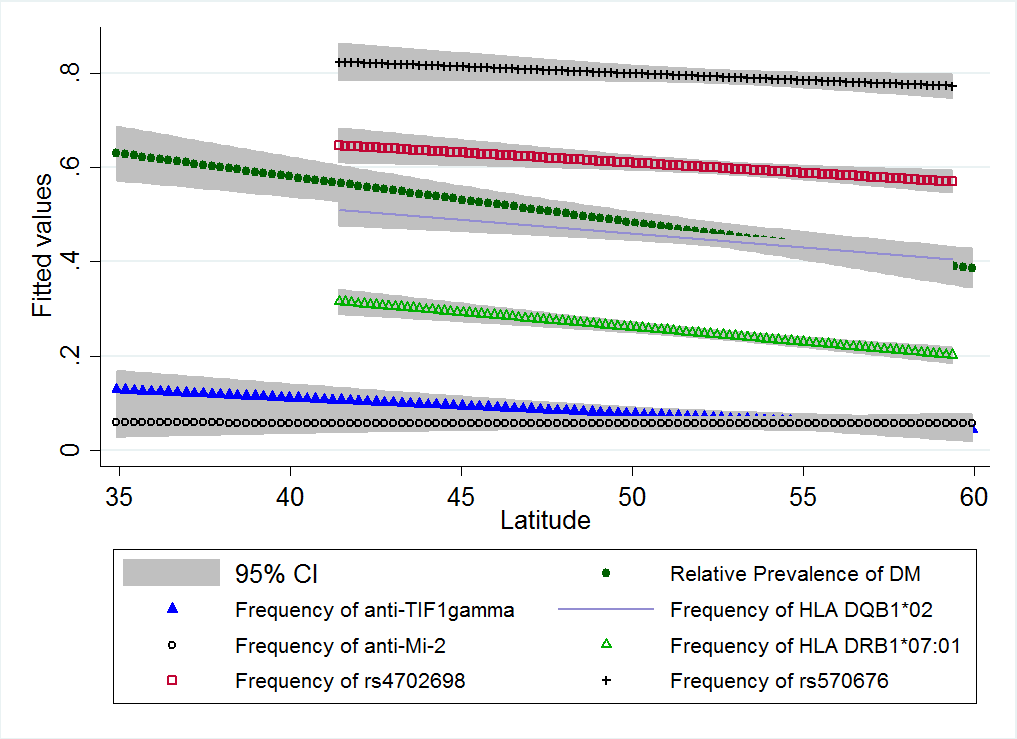

Supplement: Supplementary file 2 — Figure S1. The association of relative dermatomyositis (DM) prevalence, frequency of DM-associated autoantibodies, HLA alleles and single-nucleotide polymorphisms with latitude. Two-way linear prediction plot with 95% CIs was produced using STATA version 13.1 software. Data for DM as a proportion of PM and DM (n = 1769), frequency of anti-TIF1-γ in all samples tested (n = 1345) and frequency of anti-Mi-2 in all samples tested (n = 1471) are derived from the Immunochip study [8]. Frequencies of HLA-DQB1*02, HLA-DRB1*07:01, rs4702698 and rs570676 are derived from the Immunochip control data (n = 9911). (TIF 2203 kb) [file 13075_2018_1617_MOESM2_ESM.tif]
